# Supine vs. Prone Position With Turn of the Head Does Not Affect Cerebral Perfusion and Oxygenation in Stable Preterm Infants ≤32 Weeks Gestational Age

**DOI:** 10.3389/fphys.2018.01664

**Published:** 2018-11-22

**Authors:** Dietmar Spengler, Elisa Loewe, Martin F. Krause

**Affiliations:** Department of Pediatrics, University Hospital Schleswig-Holstein, Kiel, Germany

**Keywords:** cerebral blood flow velocity, regional cerebral oxygenation, body position, turn of head, near-infrared spectroscopy, intraventricular hemorrhage

## Abstract

Intraventricular hemorrhage (IVH) is a frequent major damage to the brain of premature babies ≤32 weeks gestational age, and its incidence (20–25%) has not significantly changed lately. Because of the intrinsic fragility of germinal matrix blood vessels, IVH occurs following disruption of subependymal mono-layer arteries and is generally attributed to ischemia-reperfusion alterations or venous congestion, which may be caused by turn of the head. Therefore, supine position with the head in a midline position is considered a standard position for preterm infants during their first days of life. We asked whether a change in body position (supine vs. prone) linked with a turn of the head by 90° in the prone position would change blood flow velocities and resistance indices in major cerebral arteries and veins of stable premature babies at two different time points (t0, day of life 2, vs. t1, day 9). Moreover, we assessed cerebral tissue oxygenation (cStO2) by near-infrared spectroscopy and determined correlations for changes in velocities and oxygenation. Twenty one premature infants [gestational age 30 (26–32) weeks] with sufficiently stable gas exchange and circulation were screened by ultrasonography and near-infrared spectroscopy. Peak systolic and end-diastolic blood flow velocities in the anterior cerebral arteries (29 ± 6 m/s vs. 28 ± 7 peak flow at t0, 36 ± 8 vs. 35 ± 7 at t1), the basilar artery, the right and the left internal carotid artery, and the great cerebral vein Galen (4.0 ± 0.8 m/s vs. 4.1 ± 1.0 maximum flow at t0, 4.4 ± 0.8 vs. 4.4 ± 1.0 at t1) did not show significant differences following change of body and head position. Also, there were no differences in cStO_2_ (83 ± 7% vs. 84 ± 7 at t0, 76 ± 10 vs. 77 ± 11 at t1) and in vital signs such as heart rate and blood pressure. We conclude that change in body position with turn of the head in the prone position does not elicit significant alterations in cerebral blood flow velocities or in oxygenation of cerebral tissues. Maturational changes in arterial flow velocities and cStO_2_ are not correlated. For this subgroup of premature infants at low risk of IVH our data do not support the concept of exclusive preterm infant care in supine position.

## Introduction

Intraventricular hemorrhage (IVH) is a frequent major damage to the brain of premature infants ≤32 weeks of gestational age (WGA)/<1500 g birthweight, and despite great advances in neonatal care its incidence (20–25%) has not significantly changed within the last two decades (Humberg et al., 2017).

Long-term neurodevelopmental outcome depends on the immaturity of the infant and the degree of parenchymal damage by IVH ranging from mild germinal matrix bleeding to widespread periventricular hemorrhagic infarction. Neurologic sequelae of IVH include all degrees of motor deficits, cognitive deficits, learning disabilities, and seizure disorders: in high-risk premature infants <1000 g birthweight Sherlock et al. (2005) found a cerebral palsy incidence of 6% in infants with grade I IVH, 24% with grade II IVH, and 100% with grade IV IVH at 8 years of age.

The risk of bleeding is inversely correlated to gestational age and may be attributed to the following three factors: vascular immaturity of the germinal matrix, deficient extravascular matrix, and cerebral blood flow disturbances (Inder et al., 2018). First, vascular immaturity bases on the observation that the germinal matrix blood vessels are composed of endothelial cells only and lack muscle tissue and collagen. This immature microvascular network contains large diameter blood vessels without arterial or venous specification, is deficient in tight junction proteins (e.g., claudin and zonula occludens protein-1), and continues to be subject to involution and remodeling until the 37th WGA (Ballabh et al., 2005; Anstrom et al., 2007). Then, the extravascular matrix is rich in fibrinolytic activity (in contrast to developmental shortfalls in platelet and coagulation factor function), but lacks direct contact with perivascular structures due to slow astrocytic development (Rao et al., 2016) making the germinal matrix a gelatinous friable tissue deficient of mesenchymal and glial elements. Finally and most important, perfusion disturbances such as fluctuating cerebral blood flow (e.g., ischemia-hyperperfusion), a pressure-passive state of the cerebral perfusion, and sudden increases in cerebral venous pressure make the premature infant subject to germinal matrix bleeding (Baburamani et al., 2012).

As the internal jugular veins are the major blood outflow tract of the brain, turn of the head associated compromise of the venous blood drainage might lead to venous congestion, increased intracranial pressure, reduced cerebral oxygenation, and ultimately germinal matrix IVH (de Bijl-Marcus et al., 2017). Therefore, the head midline position has been advocated for many years for preventing the occurrence or extension of IVH in routine neonatal care, but an updated Cochrane systematic review was unable to support this approach due to a lack of adequate studies (Romantsik et al., 2017). In routine care of the respiratory and hemodynamically stable mature infant, however, prone position is preferred for reasons of improved oxygenation (secondary to augmented functional residual capacity) (Bhat et al., 2003), saturation stability (Heimann et al., 2010), airway patency (Francois et al., 1992), drainage of oropharyngeal secretions (Pickens et al., 1989; Waisman, 2006), reduction in obstructive apnea (Heimann et al., 2010), and improved quality of sleep (Bhat et al., 2006).

Our aim was to study the impact of supine position with the head in a midline position vs. prone position with turn of the head by 90° on arterial peak and end-diastolic flow, on resistance indices in cerebral arteries, and maximum flow in cerebral veins because this practical approach to stable preterm infant nursing has not been investigated before. In addition, we assessed cerebral oxygen saturation by near-infrared spectroscopy to capture any compromise in blood supply by a supportive technique, and compared the results of two different times of examination [t0 on day 2 (median) vs. t1 on day 9].

## Materials and Methods

The detailed study protocol was approved by the institutional ethics commission of the medical faculty of the Christian-Albrechts-University, Kiel (reference number A 102/13), in accordance with the World Medical Association’s Declaration of Helsinki. The parents or the legal guardians of the infants admitted to the neonatal intensive care unit of the university hospital gave their written informed consent that could be withdrawn any time. A total of 32 premature infants ≤32 weeks of gestational age with sufficiently stable oxygenation/circulation (two infants on invasive mechanical ventilation with low pressures and FiO_2_ <0.3) in a calm resting state [grade 1–3 according to Prechtl (Cioni and Prechtl, 1990)] were recruited to avoid motion-related inaccuracies in data acquisition and sampling. Exclusion criteria following the initial screening were all conditions of low blood pressure [i.e., below the 95% confidence limit for systolic and diastolic blood pressure for age 2 and 9 days (Zubrow et al., 1995)] with or without the administration of catecholamines, large patent ductus arteriosus [defined by the following three criteria: LA:Ao-ratio of >1.6:1, ductal diameter > 2.0 mm, retrograde diastolic flow in descending Ao > 30% of forward flow (Skinner, 2000)], congenital heart disease, IVH ≥ grade 2 on an initial ultrasound examination immediately after birth, and all kinds of congenital malformations. Two infants of 27 WGA were diagnosed with unilateral IVH grade 1 on day 2 that was almost completely reabsorbed on day 9.

**FIGURE 1:**
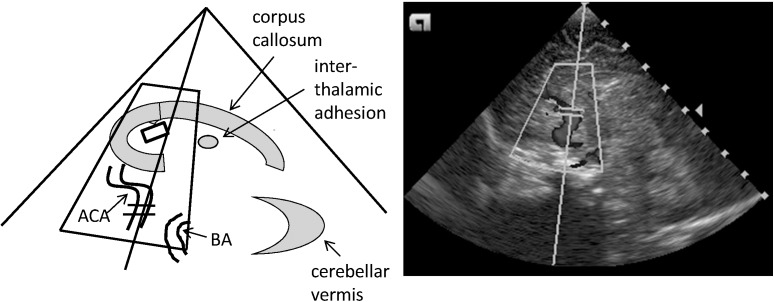
Standard sagittal plane of the cerebral color Doppler ultrasound investigation performed through the anterior fontanel. This midline sagittal image shows the corpus callosum, the interthalamic adhesion, and the cerebellar vermis as reference structures. ACA, anterior cerebral artery; BA, basilar artery.

In each infant two sets of investigations were carried out on day 2 (median, range 1–3; t0) and on day 9 (8–11; t1) of life to capture any maturational changes in all measured parameters. 6/21 infants received their t0 evaluations on day 1. The cerebral tissue oxygen saturation (cStO_2_) was assessed by the application of an infant frontal adhesive sensor to the Invos 5100C near-infrared spectroscopy device (Somanetics Corp., Troy, MI, United States). The cStO_2_ sensor was always taped to the middle of the forehead with the connecting flexible hose running to the right side. To assess cerebral perfusion an Acuson X300 (Siemens Healthcare, Erlangen, Germany) connected to a 9 MHz sector transducer was used to examine the side up anterior cerebral artery (ACA), the basilar artery (BA), the right and the left carotid artery (RICA and LICA), and the great cerebral vein Galen (GCV, the largest vessel in the midline inferior to the splenium of the corpus callosum) by sagittal and coronal sections performed via the great fontanelle as the acoustic window. For the Doppler measurements, the angle of insonation was kept <30° to reduce the error of velocity measurements and was manually corrected to a minimum (Figure 1). Color Doppler ultrasound was used to clearly identify the different blood vessels, and all recordings were cross-checked by two experienced ultrasound investigators (DS, EL). From the arterial flow profiles the peak systolic velocity (PSV) and the end-diastolic velocity (EDV) were identified, and a resistance index (RI) was calculated by PSV-EDV/PSV. Venous flow profiles of the GCV were measured in pairs using a sagittal and a coronal approach to correct for any bias in low flow venous patterns. For comparisons the maximum systolic velocities (MSV) were used to compensate for possible waveform fluctuations (Ikeda et al., 2015). The ultrasound examinations started from either the supine or the prone position without any head-up angulation, and within the examination time of 25–35 min the body position was changed (from supine with the head in a midline position to prone with a turn of the head to the right or left by 90°, or *vice versa*). The same side of head turning was used for the t0 and the t1 examinations.

We also assessed pCO_2_ (mmHg) and hemoglobin concentrations (g/dl) in capillary blood prior to each ultrasound investigation.

For statistics we applied a paired t-test to compare the data obtained from different body positions and from different time windows by the use of Prism 5 software (GraphPad, La Jolla, CA, United States). Pearson correlation of PSV and NIRS as independently measured variables were calculated. A *p*-value <0.05 was considered statistically significant.

## Results

Of the 32 preterm infants, 2 were excluded after withdrawal of informed consent; the registration of the Doppler profiles were greatly disturbed by motion-associated artifacts in 1 infant; complete sets of Doppler ultrasound recording (i.e., missing t1 recordings) were not available in two infants; in six infants the acquisition of t1 parameters could not be accomplished because of back transport to the referring hospitals. The data presented here base on the examination of 21 infants with each infant serving as its own control.

Among these 21 infants 10 (48%) were girls and 11 (52%) were boys. The gestational age was 29 ± 2 weeks, and the average weight was 1211 ± 342 g. 19/21 (90%) infants had received antenatal steroids. None of the infants was diagnosed with perinatal asphyxia (defined by an umbilical artery pH <7.1, and a 5-min Apgar score <5), even though 4/21 (19%) were prenatally screened with an absent end-diastolic umbilical artery flow. The co-morbidities are listed on Table 1. As mentioned above only infants with sufficiently stable oxygenation (SpO_2_ ≥ 92%, FiO_2_ ≤ 0.25) and circulation (blood pressure above lower 95% CL, capillary refill ≤2 s) were screened for inclusion to this study depending on the vote of the attending neonatologist. When starting cStO_2_ recordings and Doppler ultrasound examinations, the saturation by pulsed oximetry was 96% ± 2 at t0 and 97% ± 1 at t1; the heart rate was 148 bpm ± 14 at t0 and 161 ± 14 at t1; the blood pressure was 53 ± 8 mmHg (systolic)/30 ± 6 (diastolic) at t0 and 59 ± 9/35 ± 7 at t1. The results of the blood gases were: pCO_2_ 39 ± 6 mm Hg at t0 and 40 ± 4 at t1; hemoglobin 17.2 ± 2.8 g/dl at t0 and 14.6 ± 2.5 at t1.

**Table 1:** Comorbidities of the study group.

	t0^∗^ (*n* = 21)	t1 (*n* = 21)
IVH grade I^∗∗^	2 (9%)	2 (9%)
RDS^∗∗∗^	18 (85%)	11 (52%)
Large PDA^∗∗∗∗^	0	0
Apnea/bradycardia	12 (57%)	16 (76%)
Infection (antibiotic use)	15 (71%)	3 (14%)


*^∗^t0 examinations on day 2 (median, range 1–3); t1 examinations on day 9 (8–11). ^∗∗^IVH, intraventricular hemorrhage; infants with IVH ≥ grade II were excluded from the study. ^∗∗∗^RDS, respiratory distress syndrome, mild to moderate; two infants received invasive mechanical ventilation at t0. ^∗∗∗∗^Large PDA (defined by LA:Ao-ratio of >1.6:1, ductal diameter >2.0 mm, retrograde diastolic flow in descending Ao > 30% of forward flow).*

**FIGURE 2:**
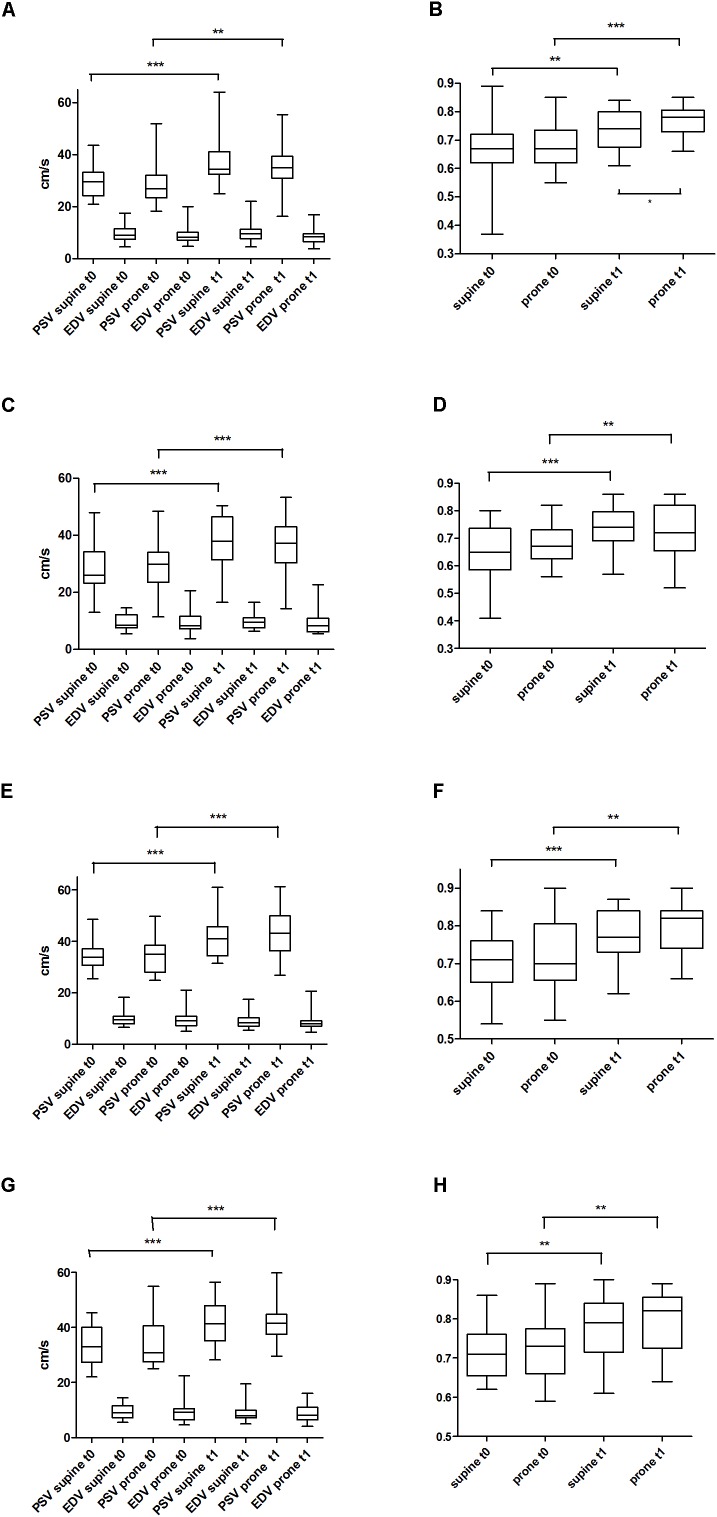
Peak systolic velocity (PSV) and end-diastolic velocity (EDV) at two different times (t0, t1) and two different body positions (supine vs. prone) of the side up anterior cerebral artery (ACA) **(A)**; resistance indices of ACA **(B)**; PSV and EDV of the basilar artery (BA) **(C)**; resistance indices of BA **(D)**; PSV and EDV of the right internal carotid artery (RICA) **(E)**; resistance indices of RICA **(F)**; PSV and EDV of the left internal artery (LICA) **(G)**; resistance indices of LICA **(H)**. ^∗^*p* < 0.05; ^∗∗^*p* < 0.01; ^∗∗∗^*p* < 0.001.

PSV, EDV, and RI from the four arteries investigated (ACA, BA, RICA, LICA) are shown in Figure 2. There were no statistically significant differences between supine and prone position at t0 and at t1 except of RI of ACA at t1 (*p* < 0.05, Figure 2B). In contrast PSV and EDV (and RI) differences of all 4 arteries between t0 and t1 suggest maturational changes and come along with an increase in blood pressure level.

**FIGURE 3:**
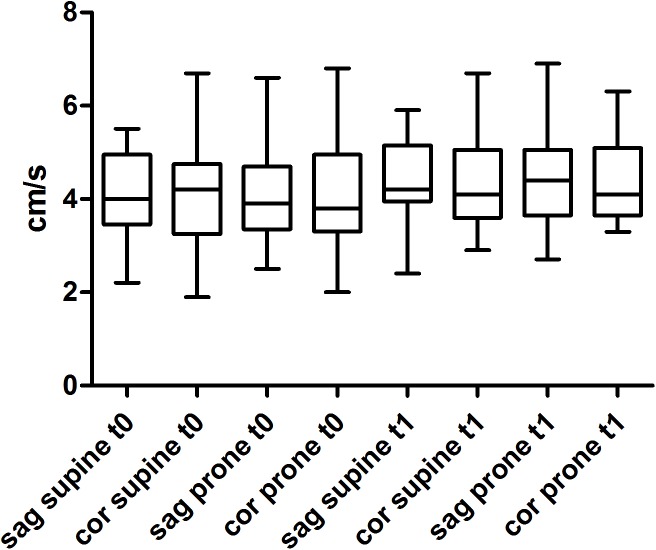
Maximum systolic flow (MSV) of the great cerebral vein (GCV) using a sagittal (sag) and a coronal (cor) approach to the anterior fontanel as an acoustic window at two different times (t0, t1) and two different body positions (supine vs. prone).

Maximum systolic velocities of the GCV was obtained by the use of two different approaches (sagittal vs. coronal) to the anterior fontanel as the acoustic window yielding no differences between supine vs. prone position, t0 vs. t1, and sagittal vs. coronal (Figure 3).

**FIGURE 4:**
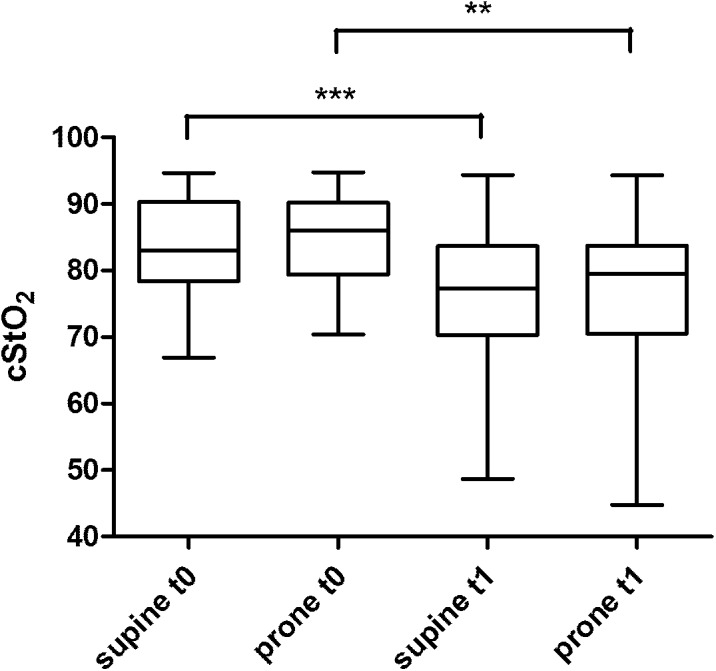
cStO_2_ (cerebral tissue oxygen saturation) recordings by NIRS technique from a frontal probe at two different times (t0, t1) and two different body positions (supine vs. prone). ^∗∗^*p* < 0.01; ^∗∗∗^*p* < 0.001.

Figure 4 displays the results of the cStO_2_ recordings without differences between supine vs. prone position, however, between t0 and t1 recordings. The calculation of correlation of PSV/EDV and cStO_2_ yielded values of *r* = 0.38 to -0.14 (not significant). Despite differences in physiologic stability in the infants with early t0 scans (i.e., day 1) the flow velocity and cStO_2_ analyses of this subgroup do not show significant differences when compared to the group examined on day 2 and 3 (data not shown).

## Discussion

Our data demonstrate that arterial and venous blood flow velocities do not depend on body position comparing supine with the head in a midline position vs. prone with a turn of the head by 90° position. In contrast we show that PSV and EDV are subject to maturational changes with increases in PSV (and RI) comparing t0 vs. t1. These changes were not observed in venous flow patterns which remained stable over time and were independent of the approach to the anterior fontanel as an acoustic window (sagittal vs. coronal). PSV and cStO_2_ were not correlated and need to be interpreted individually.

It needs to be stressed that these findings concern a subgroup of premature infants with sufficient respiratory and hemodynamic stability and may not be generalized. Infants at greatest risk of IVH typically are <28 weeks gestational age, <1000 g body weight, in their first 3–5 days of life, on invasive mechanical ventilation, equipped with umbilical lines, diagnosed with PDA or other hemodynamic instabilities, treated with specific medication for PDA closure, and often times without antenatal corticosteroids administration. Explicitly, this kind of infants was not included into this study to avoid possible undesired side effects by changes in body and head position.

However, newer technologies such as assisted non-invasive ventilation and surfactant application by different LISA (less invasive surfactant application) maneuvers allow us to provide sufficient respiratory support in a growing high-risk subgroup of premature infants as described above not receiving invasive ventilation and not being equipped with umbilical lines. Nursing in prone position with turn of the head to stabilize respiratory function is therefore mandatory and carried out in our institution beyond 96 h of age also in this subgroup of premature infants given the fact that a Cochrane Systematic Review (Romantsik et al., 2017) could not show differences in germinal matrix IVH, severe IVH, and neonatal mortality comparing supine vs. prone position.

Our findings are in line with 6 studies (Lawson et al., 1987; Buckley et al., 2009; Ancora et al., 2010; Al-Abdi et al., 2011; Elser et al., 2012; Liao et al., 2015) investigating the effect of body position (including body/head elevation) or turn of the head on oxidative phosphorylation, cerebral blood flow velocities and cStO_2_; however, the current study is the only study using both interventions simultaneously (i.e., change from supine to prone and from midline to turn of the head by 90° positions, or *vice versa*). The alteration of cerebral blood flow following turn of the head was studied by Lawson et al. (1987) in preterm and term infants using ^31^phosphorus nuclear magnetic resonance spectroscopy. The phosphocreatine/inorganic phosphate ratio did not change regardless of head-turning or supine vs. prone body position in both cohorts. Buckley et al. (2009) studied body/head elevation by 12° in 4 premature infants of 26 WGA and found no significant change in PSV, EDV, and diffuse correlation spectroscopy. They calculated a weak correlation (Pearson’s *r*^2^ = 0.13–0.58) of PSV/EDV and spectroscopy when performing body/head elevation. NIRS was used by Ancora et al. (2010) when studying 24 premature infants ≤32 WGA in six consecutive positions: supine/neutral head/bed 0°, supine/head-turning 90°/bed 0°, supine/head-turning 90°/bed + 30°, supine/neutral head/bed + 30°, prone/head minimally turned/bed + 30°, prone/head minimally turned/bed 0°. Changes in tissue hemoglobin/tissue oxygenation indices were not observed. Al-Abdi et al. (2011) assessed the incidence of IVH comparing preterm infants positioned supine with the head either in a neutral or turn of the head by 90° position and described a similar incidence (26% vs. 20%). Cerebral oxygen saturation was studied by Elser et al (Elser et al., 2012) in six different body positions involving supine/lateral/prone positions, body elevation by 15°, and turn of the head by 15–45° without any change in oxygenation (range 69–76%). Finally, Liao et al. (2015) assessed cerebral oxygen saturations in elevated supine position by 30° changing the head position several times from midline to the left/right 90° head-turning position. The range in average saturation (71–75%) was even closer as in the previous publication.

Fluctuations in the venous perfusion waveform of the GCV appear as a strong predictor of IVH in preterm infants. Ikeda et al. (2015) categorized four different GCV flow patterns in 80 VLBW infants that were repeatedly assessed over the first 6 days of life: grade 0: steady flow, constant perfusion speed; grade 1: fluctuating flow, minimum speed never less than half maximum speed; grade 2: fluctuating flow, minimum speed less than half maximum speed; grade 3: fluctuating flow, speed eventually dropping to 0 cm/s. They showed that higher grades mainly occur between days 2–4 of life which duplicates the postnatal time window of IVH occurrence; they gradually disappear beyond 4 days of life for reasons not clearly identified. Thus, the minor flow fluctuations in our recordings (grade 0 and 1 only) underline the low risk of IVH in our cohort of infants. Increased flow fluctuations in the GCV have been also described in growth retarded fetuses and are associated with an increased risk of perinatal morbidity, especially if combined with a reduction in cerebral vascular resistance and an overall increment in brain blood flow (Figueroa-Diesel et al., 2008).

The perception of head-turning related increase in intracranial pressure (ICP) and reduced cerebral perfusion by a hampered venous drainage was pursued as early as the 70th of the last century using techniques being replaced nowadays. Watson (1974) demonstrated in 1974 the complete obstruction of the ipsilateral internal jugular vein following turn of the head by 90° in anesthetized and non-anesthetized children using a contrast medium directly injected into the jugular vein. Goldberg et al. (1983) then were first to study position-related changes in ICP in preterm infants applying a Ladd monitor probe attached to the anterior fontanelle. They described a reduction [from 10.0 ± 1.2 (SEM) cm H_2_O to 6.9 ± 1.2, ns] after switching the head from a right lateral to a midline position; this effect, however, was mildly augmented in a subgroup of infants starting with a higher baseline level ICP (i.e., ≥7 cm H_2_O; from 11.9 ± 1.5 cm H_2_O to 10.0 ± 1.6, *p* < 0.01). Using the same ICP measurement technique Emery and Peabody (1983) also observed an increase in ICP by head turning [from 13.8 ± 2.5 (SEM) cm H_2_O to 17.2 ± 2.1, *p* < 0.01] along with unchanged PI (blood flow velocity parameters not provided), as was described by Urlesberger et al. (1991) in preterm infants with posthemorrhagic hydrocephalus following cancelation of head/body elevation to a horizontal position. It remains questionable whether position-related changes in ICP present a valid risk parameter for IVH as ICP depends on a manifold of factors such as cardiac output, blood pressure, cerebral perfusion, compliance of the infant skull, cerebrospinal fluid drainage, carbon dioxide level, state of vigilance, body position, stress, and gestational age (to name a few). An emerging topic is the vulnerability of the blood-brain barrier in the preterm infant following perinatal perfusion disturbances with the consequences of a delayed enwrapping of astrocytes in the germinal matrix and a reduced expression of platelet-derived growth factor beta as a key player in angiogenesis (Lai et al., 2017).

As a quality control, we compared our arterial blood flow velocity data with data from Forster et al. (2018) assessing term neonates within their first 3 days of life and found an overall consistency: ACA PSV 36.3 ± 6.6 cm/s (our data: 29.6 ± 6.2 at t0/36.8 ± 8.5 at t1); ACA EDV 12.4 ± 3.9 cm/s (9.3 ± 2.8 at t0/8.9 ± 3.3 at t1); RI 0.67 ± 0.06 (0.66 ± 0.09 at t0/0.68 ± 0.08 at t1). GCV data had to be compared to the data from Figueroa-Diesel et al. (2008) studying growth restricted fetuses: maximum flow 8.2 ± 5.5 cm/s (our data: sagittal 4.0 ± 0.8, coronal 4.1 ± 1.0 at t0). For the comparison of cStO_2_ data Fuchs et al. (2011) demonstrated a rapid rise in regional cerebral oxygenation values in preterm infants, starting at values of 35% immediately after birth and peaking within 7–10 min at 75% (our data: 83 ± 7% at t0, 76 ± 10 at t1). The slightly lower data measured in term infants may occur due to a lower oxygen extraction in the brain, a different compartment of the arterial and the venous compartment, and the registration of more central parts of the brain in preterm infants (Almaazmi et al., 2013). In addition, different devices use different types of probes to be attached to the infant’s forehead and use different algorithms for data computation. Lower cStO_2_ values of the t1 recordings may also have occurred secondary to an improved oxygen delivery to the brain parenchyma and an increased systemic blood flow (cardiac output) typically seen in neonatal transition (Vrancken et al., 2018).

There are limitations to this study concerning the small number of study patients included, the high percentage of patients excluded because of incomplete data acquisition, and the small number of extremely low birth weight infants (i.e., birthweight <1000 g, 6/21 = 28%). Around 90% of the infants received antenatal steroids, a rate in line with international publications describing large cohorts (Stichtenoth et al., 2012; Schindler et al., 2017). Major IVH is clearly linked with low gestational age, birth asphyxia, and missed administration of prenatal steroids. Indeed, our cohort of infants does not represent this subgroup of infants at highest risk for IVH. Taken together, our data demonstrate that the change from supine with the head in a midline position to prone with turn of the head by 90° position does not affect arterial or venous cerebral blood flow velocities or regional cerebral oxygenation in stable preterm infants ≤32 weeks gestational age on day 2 or day 9 of life. Cerebral blood flow and regional oxygenation are not correlated probably due to transitional hemodynamic changes, however, blood flow and regional oxygenation show strong maturational changes. Our data do not support the widespread clinical practice to nurse preterm infants in supine position with midline head position solely. Even though our findings must not be generalized for infants at high risk of IVH, a growing number of premature infants may profit by less invasive treatment protocols requiring changes in body and head position.

## Author Contributions

DS and EL performed all Doppler ultrasound examinations and supervised each other. EL collected the clinical data and performed the statistics. DS and MK designed the study. MK wrote the first draft of the manuscript. All authors approved the final manuscript.

## Conflict of Interest Statement

The authors declare that the research was conducted in the absence of any commercial or financial relationships that could be construed as a potential conflict of interest.
